# It is all about location: the performance of urgent care centers by proximity to an emergency room in a general hospital

**DOI:** 10.1186/s13584-025-00718-z

**Published:** 2025-09-18

**Authors:** Fadi Abu Saman, Liat Lerner-Geva, Zohar Mor

**Affiliations:** 1 School of Public Health, Faculty of Medicine and Health Sciences, Tel Aviv University, Tel Aviv, Israel; 2 Terem Urgent Care, Jerusalem, Israel; 3 Central Region Department of Health, Ministry of Health, Ramla, Israel

**Keywords:** Community medicine, Emergency medicine, Hospital, Emergency department

## Abstract

**Background:**

The gradual increase in referrals to the busy Emergency Departments (ED) raises concerns about the potential negative effect on the quality of medical care and patient satisfaction. Urgent Care Center (UCC) provides an alternative to the ED for non-life saving medical conditions. This study aimed to compare the admission rates, reasons for referrals and patient’s satisfaction amongst UCCs based on their distance from the nearest ED.

**Methods:**

This cross-sectional study included all medical records of four UCCs between 2017 and 2020. Admission rates, reasons for referrals, and patients' satisfaction were compared between UCCs located near and ED located farther away.

**Results:**

The study included 216,903 patients with an average age of 32.4±24.4 years. Most referrals were independent, with 37.7% occurring on weekends. The average triage time and total time spent in the UCCs were approximately 5.3 minutes and 62.8 minutes, respectively. The proportion of residents visiting UCCs in cities with an ED was 14.4% with a level of satisfaction of 91.5%, compared with UCCs in cities without ED where these figures were 23.7% and 84.4%, respectively. UCCs in northern Israel treated more patients with trauma/injury (33.0%) than UCCs in southern Israel (28.2%).

**Conclusions:**

UCCs in the two locations without an ED received a higher volume of patients, while their satisfaction levels were lower. UCC serves as an alternative to ED for non-lifesaving medical conditions.

## Background

The number of visits to emergency departments (ED) is rising, and includes a mix of medical conditions, from lifesaving to non-urgent cases [1]. To address the increasing number of referrals, various solutions have been proposed in both the hospital and ambulatory settings. In the hospital setting, solutions included increasing the number of medical staff in the ED, implementing triage at patient admission, and instituting patients’ copayments for unjustified self-referrals. In ambulatory settings, solutions included establishing telephone consultants/telemedicine, extending the operating hours and accessibility, and managing hospital referrals.

One alternative to reduce patients’ overloads in EDs was the establishment of an ambulatory urgent care center (UCC) for non-emergency cases to respond to medical conditions that are not life threatening, particularly during after-hours when community clinics are closed. Research has shown that more than 40% of the cases admitted to the ED could have been managed in an UCC [2].

The number of visits to EDs in Israel in 2020 was 2.7 million with a rate of 0.15 beds per 1,000 people. There was no significant difference in the number of visits to EDs between the northern and the southern regions in Israel (178.5/1000 and 177.3/1000, respectively) [3]. 

Terem is a chain of 25 country-wide UCCs, established in 1988 and provides off-hours medical care, including diagnostics and treatment in the community [4]. The locations of Terem UCCs are determined by their proximity to the EDs in general hospitals, amongst other administrative considerations.

In order to plan the national distribution of UCCs, this study aims to compare the rate of individuals attending UCCs, the reasons for referrals and the level of patients’ satisfaction between UCCs in cities that have an ED in a general hospital in its vicinity and UCCs in cities without an ED.

## Methods

This cross-sectional study included Terem UCCs located in four rural cities - two in the northern district and two in the southern district of Israel. For each district, one UCC was in a city with an ED, and the other was in a city without an ED (in which the nearest hospital was located at a distance of more than 30 km).

The study included all medical records of patients older than two weeks who visited one of the four UCCs between 2017 and 2020, and resided in the city in which the UCC was situated. The medical diagnoses were classified according to the ICD-10 [5]. Diagnoses that were included in block Z were excluded, as they contained ambulatory routine nursing procedures. The reasons for admission were categorized as either injury or disease.

The data for this study included demographic and medical information for each patient, the number of visits per day, the day of the visit during the week, reason for referral, medical diagnosis, time taken for patients’ triage and length of stay, level of patients’ satisfaction and discharge settings. Demographic data regarding the number of residents in each city was obtained from the statistical yearbook of the Central Bureau of Statistics (CBS) in 2019. To calculate the rate of visits to an UCC in each city, the average number of visits at each UCC during the study period was divided by the number of residents of the same city in 2019.

In order to evaluate the number of visits to the UCCs, the national average rate of visits to all Terem UCCs was calculated by dividing the total of 905,416 visits in 2019 by 9,092,000 people residing in Israel that same year [6], which was 9,923 visits per 100,000 residents. The level of satisfaction, as collected by the UCCs after each visit, was measured on a Likert scale with scores of 1 to 5. For the purpose of the study, the responses were divided as a dichotomic variable with those who were very satisfied and satisfied (levels 4 and 5) in one category, comparing them to all other responses (levels 1–3).

Independent variables including demographic, referral reason, discharge destination, time in clinic and level of satisfaction were compared between UCCs in cities with an ED and those in cities without an ED, as well as between the two clinics in the north and the two clinics in the south.

Continuous variables were compared using the Student’s *t*-test if they were normally distributed and the Mann-Whitney test if they were not normally distributed. Categorical variables were compared using chi-square test. Variables that reached statistical significance (*p* < 0.05) in the univariate analysis were included in the multivariable analysis to predict characteristics that were associated with above-average admissions per population (9,923 admissions per 100,000 residents). The results were presented as odds ratio (OR) and 95% confidence interval (CI).

## Results

**Fig. 1:**
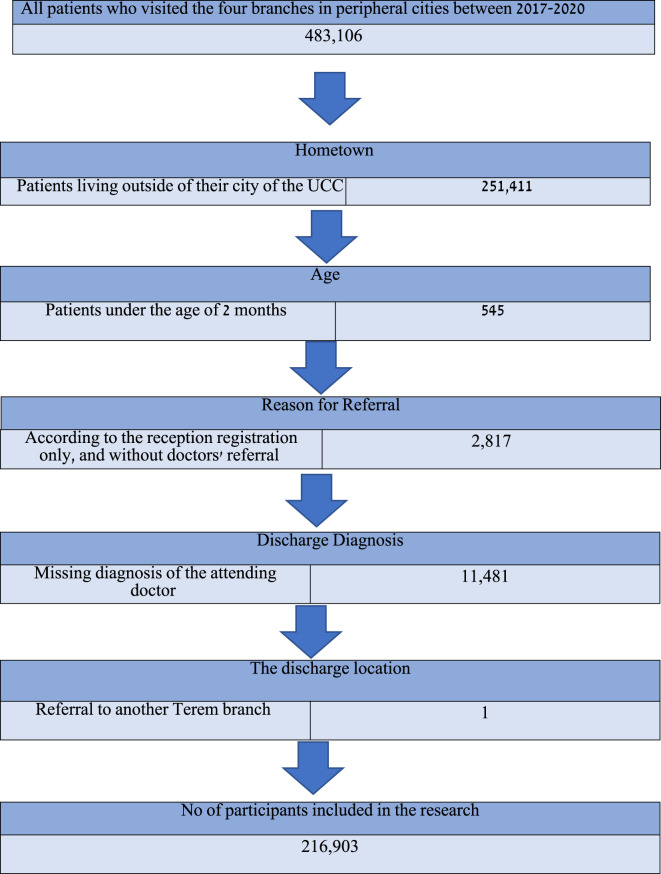
Data flow chart according to study exclusion criteria

The study included 216,903 patients who visited one of the four UCCs in the city where they resided between 2017 and 2020 (Fig. 1). Their average age was 32.4 ± 24.4 years, (*P* < 0.001), with a higher proportion of females than males (Table 1). More than one-third of all visits were during the weekend, and visits were more commonly due to injuries rather than illnesses. The waiting time for triage was 5.3 min on average, and the total average length of stay at the UCCs was 62.3 min. Most patients (91.0%, *P* < 0.001) were discharged home, and a significant majority (89.3%, *P* < 0.001) reported satisfaction with the treatment they received at the UCCs.

**Table 1:** Comparison between the four urgent care centers

Variable		Clinic 1 Southern city without hospital *N* = 61,025 (%)	Clinic 2 Southern city with hospital *N* = 87,898 (%)	Clinic 3 Northern city without hospital *N* = 38,481 (%)	Clinic 4 Northern city with hospital *N* = 29,499 (%)	TOTAL 216,903	*P*
Age (years ± SD)		22.3 ± 32.8	22.8 ± 30.2	22.4 ± 40.1	22.8 ± 34.5	24.4 ± 32.4	< 0.001
Females		31,082 (50.9%)	45,920 (52.2%)	19,701 (51.2%)	15,664 (53.0%)	112,367 (51.8%)	< 0.001
Rate of patients per 1,000 citizens out of the city population		$\:\frac{\text{15,095}}{\text{56,252}}*1000$ = 268/1000 (26.8%)	$\:\frac{\text{21,819}}{\text{142,698}}*1000$ = 153/1000 (15.3%)	$\:\frac{\text{9,483}}{\text{47,074}}*1000$ = 201/1000 (20.1%)	$\:\frac{\text{7,241}}{\text{57,741}}*1000$ = 125/1000 (12.5%)		< 0.001
Visits during weekends		23,693 (38.8%)	31,719 (36.1%)	14,485 (37.6%)	11,855 (40.2%)	81,752 (37.7%)	< 0.001
Referral for Injury/Trauma (vs. illness)		16,990 (27.8%)	64,398 (29.7%)	12,865 (33.4%)	9,543 (32.4%)	64,398 (29.7%)	< 0.001
Self-referral		51,055 (83.7%)	65,839 (74.9%)	31,746 (82.5%)	22,362 (75.8%)	171,002 (78.8%)	< 0.001
Time until triage in minutes (average ± SD)		5.1 ± 6.1	4.4 ± 5.0	5.4 ± 5.3	2.4 ± 3.1	5.1 ± 5.3	< 0.001
Length of stay at the center in minutes (average ± SD)		49.4 ± 67.6	43.8 ± 62.7	55.0 ± 74.9	40.6 ± 48.1	49.4 ± 62.8	< 0.001
Discharge destination	Discharge Home	55,404 (90.8%)	80,834 (92.0%)	34,244 (89.0%)	26,815 (90.9%)	197,297 (91.0%)	< 0.001
	Referral to hospital by ambulance	929 (1.5%)	758 (0.9%)	783 (2.8%)	175 (0.6%)	2,645 (1.2%)	< 0.001
	Referral to hospital without ambulance	4,691 (7.7%)	6,306 (7.1%)	3,554 (9.0%)	2,509 (8.5%)	16,960 (7.8%)	< 0.001
High level of satisfaction		1,567 (84.4%)	4,922 (90.7%)	1,347 (85.0%)	1,822 (93.7%)	9,658 (89.3%)	< 0.001

## Comparison between UCCs in cities with a hospital and UCCs in cities without a hospital

UCCs located in cities with a hospital treated patients at a relatively younger age, and the proportion of residents seeking medical care was approximately 1.5 times lower compared to clinics located in cities without an ED (Table 2). Self-referrals were more common in UCCs situated in cities with hospitals, and the waiting time for triage as well as the total length of stay at the UCCs were shorter.

**Table 2:** – Comparison between urgent care centers in cities with and without a hospital

Variable		Urgent care clinics in cities with an ED *N* = 117,397 (%)	Urgent care clinics in cities without an ED *N* = 99,506 (%)	*P*
Age (years ± SD)		23.9 ± 31.2	24.8 ± 33.7	< 0.001
Sex (female)		61,584 (52.5%)	50,783 (51.0%)	< 0.001
Rate of patients per 1,000 citizens out of the city population		$\:\frac{\text{29,060}}{\text{200,439}}*1000$=144/1000 (14.4%)	$\:\frac{\text{24,578}}{\text{103,326}}*1000$=237/1000 (23.7%)	< 0.001
Visits during weekends (%)		43,574 (37.1%)	38,178 (38.4%)	< 0.001
Referral for Injury/Trauma		34,543 (29.4%)	29,855 (30.0%)	< 0.001
Center referral	Self-referral	88,201 (75.1%)	82,801 (83.2%)	< 0.001
	Doctor referral	29,184 (24.9%)	16,420 (16.5%)	< 0.001
	Ambulance Referral	12 (0.1%)	285 (0.3%)	< 0.001
Time until triage in minutes (average ± SD)		4.6 4.7±	5.9 ± 6.2	< 0.001
Length of stay at the center in minutes (average ± SD)		58.9 ± 45.8	67.4 ± 52.8	< 0.001
Discharge destination	Discharge Home	107,649 (91.7%)	89,648 (90.1%)	< 0.001
	Referral to hospital by ambulance	933 (0.8%)	1,712 (1.7%)	< 0.001
	Referral to hospital without ambulance	8,815 (7.5%)	8,145 (8.2%)	< 0.001
High level of satisfaction		6,744 (91.5%)	2,914 (84.4%)	< 0.001

More patients were referred from the UCCs to the ED in cities with an ED compared to those who visited clinics situated in cities without a hospital. Conversely, patients who visited UCCs in cities with an ED were more likely to be discharged home than patients who visited UCCs in cities without an ED. UCCs in cities with an ED treated fewer trauma cases than UCCs in cities without an ED (29.4% vs. 30%, respectively, *P* < 0.001). The level of satisfaction amongst patients who visited UCCs located in cities with a hospital was greater than that of those who visited clinics in cities without a hospital.

## Comparison between two UCCs situated in the South and two UCCs situated in the North

Patients who visited southern UCCs were younger, predominantly male, with higher rates of residents from southern cities compared to those who visited the northern UCCs. Patients who visited the southern UCCs were more likely to have an illness rather than an injury, experienced longer waiting times, and reported a lower level of satisfaction (Table 3).

**Table 3:** – Comparison of urgent care centers in the North vs. centers in the South

Variable		Northern UCCs *N* = 67,980 (%)	Southern UCCs *N* = 148,923 (%)	Total 216,903 (%)	*P*
Age (years ± SD)		22.8 ± 30.2	22.3 ± 32.8	24.4 ± 32.4	< 0.001
Sex (female)		35,365 (52.0%)	77,002 (51.7%)	112,367 (51.8%)	< 0.001
Rate of patients per 1,000 citizens out of the city population		$\:\frac{\text{16,724}}{\text{104,815}}*1000$=159/1000 (15.9%)	$\:\frac{\text{36,914}}{\text{198,950}}\text{*}1000$185/1000 (18.5%)		< 0.001
Visits during weekends (%)		26,340 (38.7%)	55,412 (37.2%)	81,752 (37.7%)	< 0.001
Referral for Injury/Trauma		22,408 (33.0%)	41,990 (28.2%)	64,398 (29.7%)	< 0.001
Center referral	Self-Referral	54,108 (79.6%)	116,894 (78.5%)	171,002 (78.8%)	< 0.001
	Doctor Referral	13,611 (20.0%)	31,993 (21.5%)	45,604 (21.0%)	< 0.001
	Ambulance Referral	261 (0.4%)	36 (0.1%)	291 (0.1%)	< 0.001
Time until triage in minutes (average ± SD)		5.1 ± 4.7	5.6 ± 5.5	5.3 ± 5.1	< 0.001
Length of stay at the center in minutes (average ± SD)		52.9 ± 63.1	47.6 ± 62.7	49.4 ± 62.8	< 0.001
Discharge destination	Discharge home	61,059 (89.8%)	136,238 (91.5%)	197,297 (91.0%)	< 0.001
	Referral to hospital via ambulance	958 (1.4%)	1,687 (1.1%)	2,645 (1.2%)	< 0.001
	Referral to hospital without an ambulance	5,963 (8.8%)	10,997 (7.4%)	16,960 (7.8%)	< 0.001
Level of satisfaction		3,169 (89.8%)	6,489 (89.1%)	9,658 (89.3%)	< 0.001

## Comparison between the patients’ level of satisfaction

Analysis of a random sample of 10,812 patients showed that a greater level of satisfaction was associated with older patients, males, those who visited the clinics on weekdays, patients who experienced shorter waiting times for triage and shorter stay at the clinic, and those who were discharged to their homes (rather than the nearest hospital) (Table 4).

**Table 4:** – Comparison between patients with high level of satisfaction to patients with low level of satisfaction

Variable		High level of patient satisfaction *N* = 9,658 (89.3%)	Low level of patient satisfaction *N* = 1,154 (10.7%)	*P*
Age (years ± SD)		33.6 ± 23.4	27.4 ± 22.7	< 0.007
Sex (female)		5,059 (52.4%)	641 (55.5%)	< 0.04
Visits during weekends		2241 (23.2%)	334 (28.9%)	< 0.001
Referral for Injury\Trauma		2976 (30.8%)	310 (26.9%)	< 0.006
Center referral	Self	7,401 (76.6%)	921 (79.8%)	< 0.02
	Doctor	2248 (23.3%)	230 (19.9%)	< 0.02
	Ambulance	9 (0.1%)	3 (0.3%)	< 0.02
Time until triage in minutes (average ± SD)		4.5 ± 4.5	6.5 ± 7.5	< 0.001
Length of stay at the center (average ± SD)		60.7 ± 45.4	79.1 ± 54.3	< 0.001
Discharge Destination	Home	9208 (95.3%)	1059 (91.8%)	< 0.001
	Referral to Hospital via ambulance	87 (0.9%)	7 (0.6%)	< 0.001
	Referral to Hospital without Ambulance	363 (3.8%)	88 (7.6%)	< 0.001

In the multivariable analysis, older age, being male, visiting the clinic on weekdays, shorter length of stay, and home discharge were variables that predicted higher levels of satisfaction (Table 5).

**Table 5:** Logistic regression to identify variables that predict patients’ satisfaction

Variable		Odds ratio (95% confidence interval)	*P*
Age (years ± SD)		1.01 (1.01–1.08)	< 0.001
Sex (male)		1.2 (1.03–1.3)	< 0.017
Visits during the week		1.3 (1.1–1.5)	< 0.001
Illness		1.1 (0.9–1.3)	< 0.07
Center referral	Self	2.4 (0.6–9.4)	< 0.2
	Doctor	2.2 (0.6–8.7)	< 0.2
	Ambulance	1	< 0.02
Length of stay at the center (average ± SD)		0.994 (0.993–0.995)	< 0.001
Discharge destination	Home	2.3 (1.8-3.0)	< 0.02
	Referral to Hospital via ambulance	2.7 (1.2–6.3)	< 0.02
	Referral to hospital without ambulance	1	< 0.02

## Discussion

Individuals seeking medical care in cities without an ED were more likely to visit UCCs than those who live in cities with an ED. Conversely, the average rate of referrals (excluding maternity cases) to the ED in the cities without an ED was lower than in cities with an ED (232 vs. 401 patients per 1,000 residents) [8]. It is likely that residents in cities without an ED found the UCCs more accessible, while residents who lived in cities with an ED had the opportunity to choose whether to refer to an UCC or to the ED in their city. It may be that individuals seeking care in cities with an ED preferred the ED over the UCC, as it is better equipped and has a wider range of medical specialties, thus being considered as more prestigious. Furthermore, the EDs in general hospitals are operating on a 24-hour basis, while some UCCs close after midnight, as shown in previous studies [4, 7]. Additionally, medical insurers implement a more liberal policy of deductibles for patients who visit UCCs clinics in cities without an ED.

The rate of individuals seeking care at UCCs due to trauma or injury was slightly lower in cities with an ED compared to residents who live in cities without an ED. This finding aligns with a previous publication [8], which reported a higher rate of admission to ED due to trauma amongst residents in cities with hospitals compared to those living in cities without an ED (115.0 vs. 53.7 patients per 1000 residents, respectively). Although UCCs provide a similar level of treatment in mild and moderate trauma or injury cases as the ED, residents living in cities with an ED preferred the ED to an UCC. It may be that the sense of urgency is greater when a patient is injured, bleeding, or has a penetrating wound, making patients choose the closest medical care setting. Additionally, patients with trauma or injury may value the level of orthopedic and surgical care offered at an ED to be of a higher standard than that offered by an UCC.

The average length of stay in UCCs located in cities without an ED was longer than that in UCCs situated in cities with an ED (67.4 ± 52.8 vs. 58.9 ± 45.8 min, respectively, *P* < 0.001). The longer treatment duration is associated with a higher patient overload in UCCs in cities without an ED. In addition, medical staff in UCCs in cities without a hospital may prefer to keep patients under medical supervision for a longer duration due to the distance to the nearest hospital. This finding is consistent with another Israeli publication, which explored the utilization profile of medical services by non-medically insured migrants [9].

Patients who visited UCCs in cities without an ED expressed lower levels of satisfaction, which may be related to the longer time spent waiting for their medical care compared to those in clinics located in cities with hospitals. This finding aligns with the results of the 2019 patient experience survey conducted by the MOH [10], which demonstrated that the level of satisfaction was inversely related to the length of stay in the hospital.

The proportion of residents seeking care in northern cities was lower compared to those in southern cities, perhaps due to the geographical distribution of hospitals in the southern region being less compatible with the population’s needs than in the northern region.

This study is subject to several limitations. First, the UCCs did not open simultaneously- some have been operating for 8 years, while others for 11 years. To reduce potential selection bias, data were collected at least one year after the last clinic was opened. Second, this study focused on four specific UCCs and may not comprehensively represent the entire spectrum of Terem UCCs across Israel. However, the long period of the study which captured all patients, provides a comprehensive perspective on the use of UCCs in the periphery. Third, an age difference was noted among individuals who attended the four clinics.

## Conclusion

This study shows that patients residing in the two rural cities without an ED utilized UCCs more frequently than those living in cities with an ED. The time spent on patients’ triage and treatment in the UCCs located in cities without an ED was longer, and the level of patient satisfaction was lower compared to UCCs in cities with an ED.

In order to reduce unjustified referrals to EDs, and also to optimize the use of available services at UCCs, especially in cities where there is a hospital, it is advisable to promote the capabilities and services provided by the UCCs among the medical teams working in the community clinics and the ambulance services.

## Recommendation

The results of this study can be used to enhance the services at the UCCs, especially in cities with an ED, to reduce unjustified referrals to the ER. First, individuals in the community should be informed about the medical capabilities of UCCs, their location and the operating times. This information can be disseminated either directly or through the insurers. Second, to reduce the co-payment of the medical services in the UCCs and to increase the co-payment for self-referrals to the ER. Third, to integrate a triage system in the ER, where nurses direct non-urgent cases to the UCCs. Fourth, ER and UCCs’ physicians can rotate between the two institutions to familiarize themselves with the different medical challenges encountered in each setting, while increasing the perceived prestige of the UCCs in the community. Additionally, a paramedical team member can be employed as a “patient liaison or patient representative” during weekends. Their role would be to explain to patients their position in the treatment process, and the importance of the tests and treatments provided during their visit.

## Data Availability

The datasets used and analysed during the current study are available from the corresponding author upon reasonable request.

## Abbreviations

UCC: Urgent Care Centers
ED: Emergency Department
MOH: Ministry of Health
CBS: Central Bureau of Statistics
